# Oral *Candida* carriage and resistance against common antifungal agents in hematopoietic stem cell transplantation recipients

**DOI:** 10.1007/s00520-024-08396-4

**Published:** 2024-02-23

**Authors:** Matti Mauramo, Nurgül Tonoz, Jörg Halter, Betsy Joseph, Tuomas Waltimo

**Affiliations:** 1 Department for Oral Health & Medicine, UZB University Centre for Dental Medicine Basel, University of Basel, Basel, Switzerland; 2 Department of Pathology, University of Helsinki and Helsinki University Hospital, HUS, Diagnostiikkakeskus, Patologia, PL 400, 00029 HUS, Helsinki, Finland; 3 Department of Hematology, University Hospital Basel, Basel, Switzerland; 4 Saveetha Dental College and Hospitals, Saveetha Institute of Medical And Technical Sciences, Chennai, India

**Keywords:** HSCT, Oral Candida, Non-albicans, Resistance

## Abstract

**Purpose:**

Allogeneic hematopoietic stem cell transplant (HSCT) recipients receiving long-term and high-dose immunosuppressive medications suffer commonly from oral candida infections. This prospective cohort study examined oral fungal carriage in HSCT recipients and screened the susceptibility against commonly used antifungal agents. An increasing oral occurrence of C*andida* spp. and the development of resistance against clinically administered fluconazole were hypothesized.

**Methods:**

Two hundred HSCT recipients were included and followed up for 2 years post-HSCT. Oral microbiological specimens were analyzed with matrix-assisted laser desorption/ionization-time of flight mass spectrometry assays (MALDI-TOF). The colorimetric method was applied for the susceptibility testing by commercially available Sensititre YeastOne (SYO®, TREK Diagnostics Systems, Thermo-Fisher, UK).

**Results:**

The prevalence of oral *Candida* spp. carriage increased statistically significantly after a year post-HSCT being 30, 26, 35, 44, and 47%, pre-HSCT, 3, 6, 12, and 24 months post-HSCT, respectively. Altogether, 169 clinical oral *Candida* strains were isolated. Fourteen *Candida* spp. were identified, and *C. albicans* was predominant in 74% of the isolates pre-HSCT with a descending prevalence down to 44% 2 years post-HSCT. An increasing relative proportion of non*-albicans* species post-HSCT was evident. No development of resistance of *C. albicans* against fluconazole was found. Instead, a shift from *C. albicans* towards non-*albicans* species, especially *C. dubliensis*, *C. glabrata*, and relatively seldom found *C. krusei*, was observed.

**Conclusion:**

Oral *Candida* carriage increases after HSCT. Instead of the expected development of resistance of *C. albicans* against fluconazole, the relative proportion of non*-albicans* strains with innate resistance against azole-group antifungals increased.

**Supplementary Information:**

The online version contains supplementary material available at 10.1007/s00520-024-08396-4.

## Introduction

*Candida* spp. occur commonly in the oral flora of healthy individuals, and their reported prevalence varies from 20 to 75% [1, 2]. Significant geographic variation is evident among cases of candidemia in different parts of the world. In a review by Falagas et al., *C. albicans* predominated in the countries of Northern Europe and Switzerland (> 60%), whereas non-albicans species seem to be predominant in Asia [3].

The occurrence of oral *Candida* may be considered as an origin of systemic infections, especially in immune-compromised patients. In a study by Odds et al., the prevalence of oral *Candida albicans* carriage was observed to be 64% among patients undergoing chemotherapy or bone marrow transplantation for hematological diseases [4, 5]. In order to prevent the spread of these commensal inhabitants into invasive fungal infections, antifungal prophylaxis, often with azole-group antifungals, is a standard of care for patients undergoing high-dose chemotherapy and subsequent hematopoietic stem cell transplantation (HSCT). Due to the prophylaxis and improvements in cancer therapies, a decrease in the incidence of invasive fungal infections has been observed among HSCT recipients [6]. Despite this, the occurrence of oral *Candida* remains high and increases during cancer treatments. In a systematic review by Lalla et al., the prevalence of oral colonization with any fungal organisms in cancer patients was observed to be 48% before treatment, 72% during treatment, and 70% after treatment when all cancer diagnoses and treatments were recorded [7].

Immunosuppression caused by cancer or its treatments affects the oral environment, particularly in terms of hyposalivation and the composition of saliva. This allows opportunistic oral pathogens, such as yeasts, to gain predominance and cause clinical infections [8]. Fungal infections of oral origin can spread directly to the gastrointestinal (GI)-tract and lungs as well as virtually all organs via the hematological spread.

Immune-compromised subjects with hematological malignancies are at high risk of invasive fungal infections [9]. Thus, fungal infections have been reported to cause substantial morbidity and even mortality among subjects with hematological malignancies [9]. This may be explained by the development of resistance during a long-term administration of azole-group antifungal agents, and/or by an increasing prevalence of non-albicans *Candida* (NAC) carriage associated with an innate resistance. In line with this, the resistance of *Candida* spp. against azole-group antifungal agents has raised alarmingly worldwide [10]. Furthermore, fungal carriage and infections caused by NAC have gained increasing attention during the past decades also among HSCT recipients [6, 11].

The present study aimed to investigate the oral *C. albicans* and NAC carriage in HSCT recipients prior to the therapy and up to 24 months post-HSCT in terms of their susceptibility status against commonly used azole-group antifungals. An increasing oral occurrence of *Candida* spp., especially NAC, and the development of resistance of *C. albicans* against fluconazole were hypothesized.

## Subjects and methods

Two hundred two consecutive adult (> 18 years) subjects reporting to the Outpatient unit of Department of Hematology, University Hospital Basel, Switzerland, prior to HSCT were examined. According to the routine treatment procedure of the Department of Hematology, the patients were referred to an oral/dental examination to the Department for Oral Health & Medicine, UZB University Centre for Dental Medicine Basel, University of Basel, Basel, Switzerland. Two out of the initial 202 patients were excluded, as one subject was under 18 years, and one rejected to participate in the study. Thus, finally, 200 patients were included.

The subjects were followed up from the beginning of January 2014 till the end of December 2016. Each participant was invited to a follow-up including pre-HSCT (0), and post-HSCT (3, 6, 12, and 24 months after HSCT). The pre-HSCT examination was carried out immediately after hospitalization, mostly prior to the start of the conditioning. During the hospitalization, the subjects were allowed to continue their normal oral hygiene habits and instructed to use antiseptic chlorhexidine mouthwash twice a day. Additionally, all the subject received azole-group (Fluconazol 400 mg × 1) antifungal prophylaxis according to the normal protocol of the Department of Hematology, University Hospital Basel, Switzerland.

### Clinical oral examinations and microbiological samples

At each time point, subjects were examined for pseudomembranous, erythematous, or hyperplastic lesions as a clinical sign of candida infection in the oral cavity. Oral swab samples were taken by rubbing the dorsal tongue and any suspicious mucosal lesions with a cotton swab. The clinical oral examinations were conducted and samples collected by two experienced clinicians professor TW and MD, DDS Adrian Ramseier.

After this, the samples were plated onto Brilliance Chromogenic *Candida* Agar plates (Oxoid, Thermo-Fisher, Switzerland). Any evidence of *Candida* colonies was sub-cultured individually using chromogenic agar with *Candida* spp. Here, specific color patterns were used for preliminary identification and the cultures were cryopreserved and stored at – 80 °C for further analyses.

### Sample preparation

After plating individual colonies onto growth medium (Sabouraud Dextrose Agar plate, Oxoid) each isolate was incubated in air at 35 °C for 24–48 h. Individual colonies were picked and transferred onto a spot of MALDI steel target plate. One microliter of formic acid (70%) was applied onto the sample, mixed, and left to air-dry at room temperature (RT). After complete drying, 1 μl of matrix solution (α-cyano-4-hydroxycinnamic acid in 50% acetonitrile and 2.5% trifluoroacetic acid) was added onto that spot and left to dry at RT. The samples were processed by inserting MALDI steel target plate into MALDI-TOF MS.

### Inoculum preparation

Each identified oral *Candida* strain was plated onto growth medium agar plates and incubated at 35 °C for 24–48 h. Isolated colonies > 1 mm in diameter were picked, emulsified in physiological saline, and homogenized by vortex. *Candida* suspensions were adjusted to match the turbidity of a 0.5 McFarland’s standard by Densimat® densitometer (BioMérieux). An inoculum of 20 μl was transferred into 11-ml tube of SYO® broth (RPMI Medium 1640 + MOPS + Supplement YO, TREK Diagnostics Systems), which resulted in the manufacture’s recommended initial cell density of 1.5 − 8 × 10^3^ CFU/ml. An inoculum of 10 μl was plated onto Sabouraud’s dextrose agar plate (Oxoid, Thermo-Fisher, Switzerland) immediately.

### Microbiological identification of the isolates, matrix-assisted laser desorption/ionization-time of flight mass spectrometry (MALDI-TOF MS) assays

The *Candida* strains were identified to a species level by MALDI-TOF MS (Microflex Bruker Daltonics, Germany). This procedure applied has been described elsewhere in detail [12]. Repeated procedure was carried out using fresh samples whenever scores < 1.7 indicating no identification were found.

### Sensitivity testing

Antifungal susceptibility testing was done by Sensititre YeastOne (SYO®, TREK Diagnostics Systems, Thermo-Fisher, UK) colorimetric method. This methodology has been described elsewhere and appears to perform well for susceptibility testing of oral yeast species [13]. The determination of MIC breakpoints was based on Clinical Laboratory Standards Institute (CLSI) 2012 M27—S4 document.

The quality control of the susceptibility testing was performed twice on the following type strains of *C. albicans* (ATCC®90029TM), *C. dubliniensis* (ATCC®MYA-646TM), *C. glabrata* (ZIB9109), *C. krusei* (ATCC®6258TM), *C. parapsilosis* (ZIB9113), and *C. tropicalis* (ATCC®750TM).

### Statistical analysis

The numbers of HSCT recipients with oral *Candida* carriage, any *Candida* spp. isolated, and resistant strains against azole-group antifungals were compared pre-HSCT and 3, 6, 12, and 24 months post-HSCT. Fischer exact and chi-square test were used to determine statistical significance and *P*-value of < 0.05 was considered statistically significant. SPSS (version 25, IBM, US) was used for statistical analysis.

## Results

### Candida species in the oral cavity

At the baseline, 200 subjects (mean age 54 years, range 18–76 years) were included in the study with 82 (41%) females and 118 (59%) males. At each study point of 3, 6, 12, and 24 months post-HSCT, the number of subjects dropped to 86, 85, 75, and 17, respectively. Diagnoses and the type of HSCT (auto- vs allogeneic) are presented in Table 1. Thirty percent of the subjects were positive for oral *Candida* occurrence at pre-HSCT with increasing prevalence up to 44% (*P* = 0.029) 12 months, and 47% (*P* > 0.05) in 24 months post-HSCT. The number of subjects with oral *Candida* carriage is presented in Table 2.

**Table 1:** The hematological diagnoses and the type of transplantation of the subjects

HSCT recipients	Gender, #		Total		HSCT	
Diagnosis	Female	Male	#	%	Autologous	Allogenous
AML	36	37	73	36.5	2	61
ALL	8	7	15	7.5	1	13
CML	2	2	4	2	-	3
CLL	2	9	11	5.5	-	10
MPNs	3	6	9	4.5	-	9
MDS	3	10	13	6.5	-	12
Anemia/ SAA	4	5	9	4.5	-	7
MM	11	24	35	17.5	22	12
Lymphoma	8	15	23	11.5	9	13
PCL	1		1	0.5	-	1
B-PLL	2		2	1	-	2
Non-hematologic disorders	2	3	5	2.5	4	1
Total #, %	82 (41%)	118 (59%)	200	100	38 (19%)	144* (72%)

Abbreviations: *AML*, acute myeloid leukemia; *ALL*, acute lymphoblastic leukemia; *CML*, chronic myeloid leukemia; *CLL*, chronic myeloid leukemia; *MPN*, myeloproliferative neoplasm; *MDS*, myelodysplastic syndrome; *SAA*, severe aplastic anemia; *MM*, multiple myeloma; *PCL*, plasma cell leukemia; *B-PLL*, B-cell prolymphocytic leukemia

**Table 2:** The number of subjects with oral *Candida* carriage pre-HSCT and 3, 6, 12, and 24 months post-HSCT

Subjects	Pre-HSCT	3 M	6 M	12 M	24 M
Female; # of *candida*/ # of subjects; (%)	23/82 (28.1%)	10/38 (26.3%)	12/38 (31.6%)	13/33 (39.4%)	4/9 (44.4%)
Male; # of *candida*/ # of subjects (%)	37/118 (31.4%)	12/48 (25.0%)	18/47 (38.3%)	20/42 (47.6%)	4/8 (50.0%)
Total; # of *candida*/ # of subjects (%)	60/200 (30.0%)	22/86 (25.6%)	30/85 (35.3%)	33/75 (44. 0%)	8/17 (47.1%)

Altogether 65 *Candida* isolates were detected pre-HSCT. *C. albicans* was the most prevalent *Candida* species with 48 positive isolates, corresponding to 74% of all isolates, and 17 (26%) NAC species were isolated. A statistically insignificant shift towards non*-albicans* species was observed a year post-HSCT, as 23 *C. albicans* isolates, corresponding to 64% of all isolates, and 13 (36%) NAC species were observed (*P* > 0.05). Two years post-HSCT, 4 *C. albicans* isolates, corresponding to 44% of all isolates, and 5 (56%) NAC species were observed (*P* > 0.05) (please see Supplementary Table 1).

Altogether, fourteen *Candida* spp. were identified. The most prevalent NAC species were *C. glabrata* and *C. dubliniensis* with 7 and 6 isolates corresponding to 41.2% and 35.3% of the NAC isolates respectively. After a year, the spectrum of NAC species was more abundant and *C. glabrata* and *C. dubliniensis* represented 23% and 15% of the NAC isolates, respectively.

### SYO® antifungal susceptibility assay

During the whole study period of 2 years, 110 *C. albicans* strains were isolated, out of which only 3 (2.7%) isolates were resistant against fluconazole. Resistant *C. glabrata* isolates against fluconazole, voriconazole, and itraconazole are shown in Table 3. All eight *C. krusei* isolates were resistant against fluconazole.

**Table 3:** SYO® antifungal susceptibility assay results of the oral isolates

*Candida* species (# of isolates)	Antifungal agent	MIC_90_ (μg/ml)	Number of resistant isolates (%)
*C. albicans* (*n*: 110)	FLU	1	3 (2.7%)
*C. glabrata* (*n*: 19)	FLU	256	4 (21.0%)
	VOR	2	19 (100%)
	ITR	8	4 (21.0%)
*C. krusei* (*n*: 8)	FLU	64	8 (100%)

Resistance (MIC_90_) is presented; *FLU*, fluconazole; *VOR*, voriconazole; *ITR*, itraconazole

## Discussion

This study examined oral fungal carriage in HSCT recipients and screened the susceptibility to commonly used antifungal agents. The oral *Candida* carriage was observed to increase statistically significantly (*P* = 0.029) within the first year post-HSCT with persisting higher prevalence during the second year. This observed increase in the prevalence of oral *Candida* carriage during the cancer therapies is in line with the results of a systematic review on oral fungal infections in patients receiving cancer therapy for solid tumors [7]. However, there are only relatively few recent studies focusing on the prevalence of *Candida* carriage among HSCT recipients being at high risk of invasive fungal infections. Together with developments in HSCT treatments and infection prophylaxis, the altered occurrence of oral yeasts in the population as well as increased resistance to azole-group antifungals, there is a need for studies of the prevalence and resistance of oral *Candida* spp. among the HSCT recipients [9, 10, 14].

In an older study by Odds et al., the prevalence of oral *Candida albicans* carriage was observed to be 64% in patients prior to HSCT [4]. In the present study, the prevalence of oral *Candida* carriage was lower, with raising prevalence from the initial pre-HSCT state of 30% up to 44% a year and 47% 2 years post-HSCT. In another European study, the oral *Candida* prevalence of 63.9% was observed among HSCT recipients’ pre-transplantation [15]. In a study by Shirazian from Tehran, Iran (2020) among subjects with acute myeloid leukemia (AML), the prevalence of oral *Candida* colonization was observed in 41.2% of the patients more frequently compared with healthy subjects (38.7%) [16]. However, the methods of oral sampling and analysis of *Candida* spp. can vary remarkably between the studies. In a study by Laheij et al., oral samples were taken at least twice a week starting before or as soon as possible after the administration of the conditioning regimen and continued until hospital discharge. All patients had samples that were positive for at least one *Candida* spp. during the course of the study [17].

Among hematological patients, *Aspergillus* spp. and *Candida* spp. cause most cases of invasive fungal infections [9]. However, the use of routine prophylaxis for patients at high risk for invasive fungal infections has caused a shift in epidemiology, in particular to NAC spp. such as *C. glabrata*, *C. krusei*, and *C. tropicalis* [9]. In the current study, the most prevalent NAC species were *C. glabrata* followed by *C. dubliniensis* with 41.2% and 35.3% of the NAC isolates, respectively. A tendency towards NAC species was observed although statistically insignificant. However, it should be pointed out that the second most common species, *C. dubliniensis*, is morphologically and phylogenetically very similar to *C. albicans*. It has been shown to differ from *C. albicans* in genes coding for virulence-associated proteins and *C. dubliniensis* lacks more than 168 genes characteristic of *C. albicans* [18]. Nonetheless, increased prevalence of NAC spp. among leukemia patients and patients receiving HSCT has also been reported in other studies. In the study by Shirazian et al., *Candida glabrata* was the most prevalent *Candida* spp. among AML subjects [16]. In the European study by Laheij et al., in line with the current study, *C. albicans* was the most prevalent species, but a high prevalence of NAC species *C. glabrata*, *C. tropicalis*, and *C. krusei* were also detected, with an oral carriage of 70%, 80%, and 67%, respectively [17]. In addition to geographic variation, there is also a clear difference in the prevalence of oral *Candida* carriage according to cancer types. In a recent Finnish study among oral cancer patients, an oral *Candida* prevalence of 75% was observed with *C. albicans* being the most common *Candida* spp. [19]. The NAC strains were present in 16% of the pre-operative samples and 14% of the follow-up samples (follow-up: minimum of 19 months) [19].

There has been an increase in candida infections worldwide with increasing resistance to commonly used azole-group antifungal agents [10]. In this study, a development of resistance of *C. albicans* against fluconazole was hypothesized. However, contrary to the hypothesis this was not evident. Throughout the whole study period of 2 years, 110 *C. albicans* strains were isolated, out of which only 3 (2.7%) isolates were resistant against fluconazole with random distribution within the time-points of the study. Similarly, no increase in resistance against fluconazole or itraconazole was observed in *C. glabrata.* However, 100% of *C. krusei* isolates were resistant against fluconatsole and 100% of *C. glabrata* isolates against voriconazole.

Mann et al*.* studied the effect of the azole antifungal agents (posaconazole, fluconazole, or itraconazole) on susceptibility of *Candida* in patients receiving allogeneic bone marrow transplantation or with acute myeloid leukemia [11]. The relative occurrence of *C. glabrata* was increased two- to fourfold when patients were prescribed posaconazole and itraconazole, respectively, and that of *C. krusei* doubled during systemic fluconazole administration. The strains of *C. glabrata* isolated from the first sampling multiplied their azole minimum inhibitory concentrations by a factor of 4 at the end of antifungal treatment [11].

The results of the current study, opposing our hypothesis of the development of resistance by *C. albicans* and partly contrasting previous study reporting resistance development by NAC, may have several explanations. Relative increase in the proportion of NAC with partially innate resistance against azole-group antifungal agents was observed during the study and no development of resistance during the follow-up concerning individual *Candida* species was observed. Furthermore, the concentration of azoles in oral fluids, saliva, and gingival crevicular fluid may be too low to trigger resistance in oral *Candida* which may survive in organized oral biofilm, providing additional protection from adverse environmental conditions [5]. Thus, *Candida* spp. in other anatomic locations, particularly in lower GI- and urinary track, may possess different susceptibility for resistance development, warranting further studies.

There was a significant loss of subjects within the observation period affecting the reliability of the study. Due to death, referrals to other hospitals and dropouts, the number of subjects particularly 2-year post-HSCT was remarkably reduced. Thus, the findings must be treated with caution. Furthermore, the pre-HSCT prevalence of oral yeast carriage should be interpreted with care due to the variable course of illness and therapy prior to the HSCT, previous oral hygiene, and dietary habits. In particular, the use of antiseptic oral rinses, such as chlorhexidine, routinely prescribed for the maintenance of oral hygiene during the conditioning therapy may have affected the results in some patients. However, the vast majority of the pre-HSCT samples was collected prior to the conditioning. Thus, studies with more subjects in more centralized follow-up set-ups would allow statistical analyses with significant results concerning the development of resistance and the presented tendency of shift from the oral carriage of *C. albicans* towards NAC species. Additionally, confounding factors predisposing to candida infections should be considered including oral hygiene measurements, saliva flow rate, oral mucositis, and systemic conditions, such as diabetes.

In conclusion, oral *Candida* carriage seems to increase after HSCT. Instead of the development of resistance of *C. albicans* against fluconazole, the relative proportion of non*-albicans* strains with innate resistance against azole-group antifungals seems to increase. This should be considered in prophylaxis and when treating oral or systemic fungal infections among HSCT recipients.

### Supplementary Information

Below is the link to the electronic supplementary material.

Supplementary file1 Supplementary Table 1. Distribution, number of isolates, and percentage of the non-albicans species. (DOCX 13 KB)

## Data Availability

The data of the study is available upon request form the corresponding author.
