# Supplementary material for: Oral Candida carriage and resistance against common antifungal agents in hematopoietic stem cell transplantation recipients
Source: Support Care Cancer. 2024 Feb 23;32(3):185. doi: 10.1007/s00520-024-08396-4 (PMC10891237; doi:10.1007/s00520-024-08396-4)
Supplement: Supplementary file 1 — Supplementary file1 Supplementary Table 1. Distribution, number of isolates, and percentage of the non-albicans species. (DOCX 13 KB) [file 520_2024_8396_MOESM1_ESM.docx]

**Supplementary Table 1.** Distribution, number of isolates, and percentage of the non-albicans species.

| Distribution of non-albicans spp. | Pre-HSCT (n=17) | 3 M (n=9) | 6 M (n=14) | 12 M (n=13) | 24 M (n=5) |
| --- | --- | --- | --- | --- | --- |
| *C. dubliniensis* | 6 (35.3%) | 1 (11.1%) | 1 (7.1%) | 2 (15.4%) | 2 (40.0%) |
| *C. glabrata* | 7 (41.2%) | 2 (22.2%) | 6 (42.8%) | 3 (23.1%) | 1 (20.0%) |
| *C. krusei* | 1 (5.9%) | 3 (33.3%) | 2 (14.3%) | 1 (7.7%) | 1 (20.0%) |
| *C. parapsilosis* | 2 (11.8%) | 0 (0%) | 1 (7.1%) | 0 (0%) | 0 (0%) |
| *C. kefyr* | 1 (5.9%) | 0 (0%) | 1 (7.1%) | 1 (7.7%) | 0 (0%) |
| *C. guilliermondii* | 0 (0%) | 1 (11.1%) | 1 (7.1%) | 0 (0%) | 1 (20.0%) |
| *C. lusitaniae* | 0 (0%) | 1 (11.1%) | 1 (7.1%) | 1 (7.7%) | 0 (0%) |
| *C. intermedia* | 0 (0%) | 1 (11.1%) | 0 (0%) | 0 (0%) | 0 (0%) |
| *C. tropicalis* | 0 (0%) | 0 (0%) | 1 (7.1%) | 1 (7.7%) | 0 (0%) |
| *C. orthopsilosis* | 0 (0%) | 0 (0%) | 0 (0%) | 1 (7.7%) | 0 (0%) |
| *C. lambica* | 0 (0%) | 0 (0%) | 0 (0%) | 1 (7.7%) | 0 (0%) |
| *C. sphaerica* | 0 (0%) | 0 (0%) | 0 (0%) | 1 (7.7%) | 0 (0%) |
| *C. pararugosa* | 0 (0%) | 0 (0%) | 0 (0%) | 1 (7.7%) | 0 (0%) |
